# Necrotizing enterocolitis in very preterm infants: an epidemiological study in Jiangsu, China

**DOI:** 10.3389/fped.2026.1948550

**Published:** 2026-09-23

**Authors:** Yan Guo, Xu Chen, Jingai Zhu, Shifeng Lu, Song Liu, Bo Wang, Rui Cheng, Aimin Qian

**Affiliations:** 1 Department of Neonatology, The Affiliated Jiangning Hospital of Nanjing Medical University, Nanjing, China; 2 Department of Neonatology, Children’s Hospital of Nanjing Medical University, Nanjing, China; 3 Department of Pediatrics, Women’s Hospital of Nanjing Medical University, Nanjing, China; 4 Department of Pediatrics, Nanjing Women and Children’s Healthcare Hospital, Nanjing, China; 5 Department of Hematology, Children’s Hospital of Nanjing Medical University, Nanjing, China; 6 Department of Pediatrics, Nanjing Pukou People’s Hospital, Liangjiang Hospital, Southeast University, Nanjing, China; 7 Department of Pediatrics, The Affiliated Suqian Hospital of Xuzhou Medical University, Suqian, China; 8 Department of Pediatrics, The Affiliated Suqian First People’s Hospital of Nanjing Medical University, Suqian, China

**Keywords:** China, clinical practices, epidemiology, necrotizing enterocolitis, very preterm infants

## Abstract

**Background:**

Epidemiological data focusing on clinical practices for necrotizing enterocolitis (NEC) in China remains scarce. To characterize the incidence, clinical management, and outcomes of NEC among very preterm infants (VPIs) in China.

**Methods:**

This study included all VPIs (gestational age <32 weeks) admitted to seven hospitals in Jiangsu, China between 2019 and 2021. Infants who died or were discharged within 72 h were excluded. NEC was defined as Bell's stage ≥ II.

**Results:**

Among 3,088 included infants, NEC incidence was 5.1% (156/3,088), decreasing with advancing gestational age: 7.7% at <28 weeks, 5.8% at 28–29 weeks, and 3.7% at 30–31 weeks. The rates of key perinatal/neonatal practices were 78.1% for antenatal steroids, 17.7% for delayed cord clamping, and 52.1% for human milk feeding. Among 156 NEC cases, NEC mortality was 16.7% (26/156), peaking at 30% in <28w group. Median nil per os duration was 8 (IQR: 5–11) days and time to full feeds was 25 (IQR: 14–38) days post-onset. Only 18.0% were initiated any human milk feeding after the NEC-related fasting period. Median antibiotic duration for NEC was 17 (IQR: 11–26) days, and 51.9% of cases received carbapenems.

**Conclusions:**

Overall NEC incidence and mortality in VPIs were comparable to high-income country benchmarks; however, the 28-31 week subgroup showed higher incidence than these benchmarks, whereas the <28 week subgroup showed higher mortality. These findings highlight the importance of gestational-age-stratified interpretation and may inform future quality improvement efforts in perinatal care, human milk feeding, and antibiotic stewardship.

## 1 Introduction

Necrotizing enterocolitis (NEC) is a severe, destructive inflammatory bowel disease associated with high mortality, prolonged hospitalization, and substantial disease burden, making it one of the most critical prematurity-related complications in very preterm infants (VPIs, < 32 weeks' gestational age) (1, 2). Recent studies have reported significant variations in NEC incidence and clinical management across different ethnicities and economic settings, with notable disparities between high- and low-income countries (3, 4). Globally, the reported incidence of NEC in VPIs ranges from 2% to 7% in high-income countries (3, 5–7), while the incidence in China is 4.8% (8).

A comprehensive assessment of NEC's disease burden, clinical features, and current management practices is essential for identifying potential areas for quality improvement and informing preventive strategies. However, limited studies have systematically investigated the epidemiology, management strategies, or short-term outcomes of NEC in VPIs in China. Therefore, we conducted a multicenter observational study to characterize the burden, gestational-age-specific epidemiological patterns, clinical management practices, and short-term outcomes of NEC among VPIs in China, using a regional cohort from seven Chinese Neonatal Network (CHNN)-participating hospitals. Our aim was to provide multicenter regional data on NEC among VPIs in China and to identify potential areas for quality improvement in NEC prevention and care.

## 2 Materials and methods

### 2.1 Data source

This multicenter retrospective cohort study utilized data from the CHNN database, specifically extracting records from seven participating hospitals in Jiangsu Province. The CHNN has maintained a standardized national clinical database for preterm infants < 32 weeks' gestation or < 1,500 g admitted to tertiary neonatal intensive care units (NICUs) throughout China. Established on January 1, 2019, this prospective database monitors trends in clinical outcomes and care practices to identify potential strategies for improving neonatal care (9). Trained personnel extracted patient information from medical records using predefined standardized definitions. Data were entered into a customized electronic system with built-in error-checking functions before being securely transmitted to the CHNN Coordinating Center at the Children's Hospital of Fudan University. All data were de-identified to ensure confidentiality. The center performed automated plausibility checks, manual verification for completeness/accuracy, and regular audits to uphold data integrity (10).

### 2.2 Sites

The study encompassed seven tertiary-level NICUs distributed across five major cities in Jiangsu, China. The participating institutions included: four maternal and child health hospitals, two freestanding children's hospitals, and one general hospital. All hospitals were provincial-level critical neonatal care centers with capacity to provide comprehensive care for infants < 28 weeks' gestation or < 1,000 g birth weight, including three centers offering neonatal general surgery services. Data were collected from the seven NICUs in Jiangsu Province and may not fully represent the national epidemiology of NEC in China.

### 2.3 Study population

We included all VPIs (< 32 weeks' gestation) admitted to the seven CHNN-participating NICUs in Jiangsu between January 1, 2019, and December 31, 2021. Infants who died or were discharged against medical advice within 72 h of birth were excluded, as none were diagnosed with NEC before death or discharge within 72 h. NEC status after such early death or discharge could not be reliably ascertained from the available data, and a sensitivity analysis was therefore not feasible. Follow-up continued until death or NICU discharge.

### 2.4 Definitions

NEC was defined as stage ≥ II according to Bell's stage (11). NEC-related death was defined as death occurring within 7 days of NEC onset. Surgical NEC was defined as cases requiring laparotomy with or without intestinal resection, enterostomy, or peritoneal drain placement. Gestational age was determined using a hierarchy of best obstetric estimates, including prenatal ultrasound, menstrual history, and obstetric examination. If obstetric estimates were unavailable or differed from postnatal assessments by > 2 weeks, gestational age was calculated using the Ballard score (12). Antenatal steroids was defined as administration of at least one partial or complete course of corticosteroids prior to delivery. Chorioamnionitis was diagnosed clinically or histopathologically. Transport Risk Index of Physiologic Stability (TRIPS) was a validated neonatal illness severity score at NICU admission, comprising four physiologic variables: temperature, blood pressure, respiratory status, response to noxious stimuli (13). Early-Onset Sepsis was defined as culture-confirmed sepsis within 72 h of birth. Human milk feeding referred to the receipt of mother's own milk or donor milk. Inotropes used included the use of dopamine, dobutamine, norepinephrine, vasopressin, epinephrine, or milrinone. Patent ductus arteriosus treatment referred to either medical therapy (acetaminophen, indomethacin, ibuprofen) or surgical ligation. Periventricular leukomalacia was defined as the presence of cystic lesions in periventricular white matter on cranial ultrasound or MRI (14). Retinopathy of Prematurity (ROP) was classified according to the International Classification of ROP (15). Bronchopulmonary dysplasia (BPD) was defined as oxygen or ventilator dependence at 36 weeks' postmenstrual age, or at discharge or death if before 36 weeks (16). Small for Gestational Age (SGA) was defined as birth weight < 10th percentile for gestational age and sex, based on Chinese neonatal growth standards (17). Extrauterine growth restriction was defined as discharge weight < 10th percentile for corrected gestational age (per 2013 Fenton growth curves) or weight < -2SD at 50 weeks' postmenstrual age (WHO Child Growth Standards) (18, 19).

### 2.5 Statistical analysis

Data were expressed as frequencies (%) for categorical variables and medians (IQRs) for continuous variables. Group comparisons were performed using *χ*^2^ tests or Mann–Whitney *U* tests, respectively, based on variable type. Trend analyses across gestational age, birth weight, and time periods utilized Cochran-Armitage tests (binary variables) and Jonckheere–Terpstra tests (continuous variables) to evaluate ordered associations. Univariate and multivariate generalized estimating equations (GEE) models were used to estimate the odds ratios for outcomes between NEC and non-NEC cases, as well as between medically and surgically managed NEC patients. The multivariate analysis adjusted for patient-level confounders including gestational age, sex, multiple births, SGA, inborn status, 5-minute Apgar score, TRIPS score, antenatal steroid administration, and prolonged rupture of membranes (> 24 h), while accounting for center clustering effects. For outcomes with missing data, analyses were performed using available cases, with no imputation of missing outcome data. The corresponding denominators are reported for each outcome. Analyses were performed using SAS 9.4 (SAS Institute, Cary, NC). Two-tailed tests were applied, with *P* < 0.05 considered statistically significant.

## 3 Results

### 3.1 Infant, maternal, and clinical characteristics

A total of 3,175 VPIs were admitted during the study period. After excluding 87 infants who died or were discharged within 72 h of birth, 3,088 infants were included in the analysis (Supplementary Figure 1). The cohort was 56.2% (1736/3088) male, with median birth weight of 1,320 g (IQR: 1,100–1,550; range: 440–2,400) and gestational age of 30.0 weeks (IQR: 28.4–31.0; range: 22.7–31.9). The rates of key perinatal and neonatal practices were 78.1% (2411/3088) for antenatal corticosteroid administration, 17.7% (545/3088) for delayed cord clamping, 52.1% (1608/3088) for human milk feeding initiation (Table 1).

**Table 1:** Infant, maternal, and clinical characteristics of very preterm infants with NEC and non-NEC.

Variables	All infants (*n* = 3,088)	Non-NEC (*n* = 2,932)	NEC (*n* = 156)	*P* value^a^
Infant characteristics				
Gestational age, weeks, median (IQR)	30.0 (28.4–31.0)	30.0 (28.4–31.0)	29.0 (27.9–30.1)	<0.001
Birth weight, g, median (IQR)	1,320 (1,100–1,550)	1,330 (1,100–1,550)	1,200 (1,000–1,420)	<0.001
Male, *n* (%)	1,736 (56.2)	1,643 (56.0)	93 (59.6)	0.380
Multiple births, *n* (%)	872 (28.2)	822 (28.0)	50 (32.1)	0.278
SGA, *n* (%)	255 (8.3)	234 (8.0)	21 (13.5)	0.015
Inborn, *n* (%)	2,084 (67.5)	1,994 (68.0)	90 (57.7)	0.007
5 min Apgar ≤3, n/N (%)	37/3,043 (1.2)	35/2,893 (1.2)	2/150 (1.3)	0.893
TRIPS score, median (IQR)	11 (6–14)	11 (6–14)	12 (6–19)	0.047
Maternal characteristics				
Maternal age <18 or >35, years, *n* (%)	462 (15.0)	441 (15.0)	21 (13.5)	0.590
Cesarean, *n* (%)	1,646 (53.3)	1,563 (53.3)	83 (53.2)	0.980
Antenatal steroids, *n* (%)	2,411 (78.1)	2,297 (78.3)	114 (73.1)	0.121
Antenatal MgSO4, *n* (%)	1,158 (37.5)	1,109 (37.8)	49 (31.4)	0.107
Delayed cord clamping, *n* (%)	545 (17.7)	518 (17.7)	27 (17.3)	0.909
Cord milking, *n* (%)	929 (30.1)	890 (30.4)	39 (25.0)	0.155
Assisted Reproduction, *n* (%)	478 (15.5)	448 (15.3)	30 (19.2)	0.184
Cervical Sutures, *n* (%)	98 (3.2)	93 (3.2)	5 (3.2)	1.000
HDP, *n*/*N* (%)	587/3,069 (19.1)	553/2,914 (19.0)	34/155 (21.9)	0.362
GDM, *n*/*N* (%)	559/3,068 (18.2)	528/2,913 (18.1)	31/155 (20.0)	0.556
PROM >24 h, *n*/*N* (%)	733/2,982 (24.6)	708/2,834 (25.0)	25/148 (16.9)	0.026
Chorioamnionitis, *n* (%)	522 (16.9)	497 (17.0)	25 (16.0)	0.764
Clinical characteristics				
Human milk feeding initiation, *n* (%)	1,608 (52.1)	1,529 (52.2)	79 (50.6)	0.713
Age at feeding initiation, days, median (IQR)	1 (1–3)	1 (1–3)	2 (1–7)	0.006
Age at first full enteral feeding, days, median (IQR)^b^^,^ ^c^	18 (12–29)	18 (12–28)	26 (16–44)	<0.001
Invasive ventilation, *n* (%)^c^	1,144 (37.1)	1,042 (35.5)	102 (65.4)	<0.001
UVC, *n* (%)	1,392 (45.1)	1,311 (44.7)	81 (51.9)	0.078
UAC, *n* (%)	53 (1.7)	46 (1.6)	7 (4.5)	0.016
PICC, *n* (%)	1,692 (54.8)	1,585 (54.1)	107 (68.6)	<0.001
Caffeine, *n* (%)	2,408 (78.0)	2,275 (77.6)	133 (85.3)	0.024
PDA treatment, *n* (%)	362 (11.7)	336 (11.5)	26 (16.7)	0.049
Red blood cell transfusions, *n* (%)^c^	1,726 (55.9)	1,589 (54.2)	137 (87.8)	<0.001
Inotropes used, *n* (%)^c^	1,117 (36.2)	1,021 (34.8)	96 (61.5)	<0.001
Early-onset sepsis, *n* (%)	29 (0.9)	28 (1.0)	1 (0.6)	1.000
Early antibiotics within first 7 postnatal days				
Any antibiotic use, *n* (%)	2,754 (89.2)	2,621 (89.4)	133 (85.3)	0.105
≥ 2 antibiotic agents, *n*/*N* (%)^d^	1,532/2,754 (55.6)	1,440/2,621 (54.9)	92/133 (69.2)	0.001
Antibiotic duration >3 days, *n*/*N* (%)^d^	2,559/2,754 (92.9)	2,432/2,621 (92.8)	127/133 (95.5)	0.236
Carbapenem use, *n*/*N* (%)^d^	442/2,754 (16.0)	412/2,621 (15.7)	30/133 (22.6)	0.036

NEC, necrotizing enterocolitis; GA, gestational age; IQR, interquartile range; SGA, small for gestational age; TRIPS, transport risk index of physiologic stability; HDP, hypertensive disorders of pregnancy; GDM, gestational diabetes mellitus; PROM, premature rupture of membranes; UVC, umbilical vein catheterization; UAC, umbilical artery catheterization; PICC, peripherally inserted central catheter; PDA, patent ductus arteriosus.

a All *P* values represent comparisons between NEC and non-NEC groups.

b Calculated in 2,821 surviving infants (Non-NEC 2,691, NEC 130).

c These are descriptive in-hospital course measures and may reflect post-onset care, and are therefore not antecedent risk factors.

d Among infants receiving antibiotics within the first 7 postnatal days.

Significant differences were observed between NEC and non-NEC groups (Table 1). Infants with NEC had lower gestational age, lower birth weight, higher rates of SGA, more frequent outborn status and slightly higher TRIPS scores. Enteral feeding was also initiated later in the NEC group. Early antibiotic exposure within the first 7 postnatal days was similar between the NEC and non-NEC groups; however, among infants receiving antibiotics, those in the NEC group more frequently received two or more antibiotic agents and carbapenems, while the antibiotic duration was similar. Importantly, time to full enteral feeding, invasive ventilation, red blood cell transfusion, and inotropic support were in-hospital course measures that may have occurred after NEC onset. These variables may therefore reflect both underlying illness severity and post-NEC clinical management and should not be interpreted as antecedent risk factors for NEC.

### 3.2 Incidence and clinical characteristics of NEC

The overall incidence of NEC was 5.1% (156/3088), with stage ≥ III NEC occurring in 1.5% (46/3088). The median age at NEC onset was 18.0 (IQR: 10.0–28.5) days. Among 156 NEC cases, intestinal perforation was observed in 17.3% (27/156) and surgical intervention required in 31.4% (49/156) (Table 2).

**Table 2:** NEC in very preterm infants: incidence and clinical characteristics of NEC.

Group	All infants, *N*	NEC, *n* (%)	Stage Ⅲ NEC, *n* (%)	Age at NEC onset, days, median (IQR)	Intestinal perforation, *n* (%)^a^	Surgical treatment, *n* (%)^a^	Death, *n* (%)^a^	NEC-related death, *n* (%)^a^^,^^b^
Total	3,088	156 (5.1)	46 (1.5)	18.0 (10.0–28.5)	27 (17.3)	49 (31.4)	26 (16.7)	19 (12.2)
Gestational age group								
<28weeks	522	40 (7.7)	13 (2.5)	23.0 (13.0–31.5)	7 (17.5)	16 (40.0)	12 (30.0)	7 (17.5)
28–29 ^+^ ^6^ weeks	1,012	59 (5.8)	16 (1.6)	23.0 (14.0–34.0)	9 (15.3)	20 (33.9)	9 (15.3)	8 (13.6)
30–31 ^+^ ^6^ weeks	1,554	57 (3.7)	17 (1.1)	12.0 (6.0–18.0)	11 (19.3)	13 (22.8)	5 (8.8)	4 (7.0)
*Z* value		3.856^c^	2.257^c^	−3.984^d^	−0.287^c^	1.842^c^	2.705^c^	1.589^c^
*P* for trend		<0.001	0.024	<0.001	0.774	0.066	0.007	0.112
Birth weight group								
<1,000 g	459	38 (8.3)	15 (3.3)	25.5 (14.0–39.0)	9 (23.7)	19 (50.0)	8 (21.1)	7 (18.4)
1,000–1,499 g	1,649	87 (5.3)	23 (1.4)	19.0 (9.0–28.0)	12 (13.8)	25 (28.7)	15 (17.2)	9 (10.3)
≥1,500 g	980	31 (3.2)	8 (0.8)	12.0 (8.0–15.0)	6 (19.4)	5 (16.1)	3 (9.7)	3 (9.7)
*Z* value		4.139^c^	3.315^c^	−4.105^d^	0.570^c^	3.068^c^	1.241^c^	1.161^c^
*P* for trend		<0.001	<0.001	<0.001	0.568	0.002	0.215	0.246
Year group								
2019	886	63 (7.1)	17 (1.9)	14.0 (6.0–29.0)	12 (19.0)	19 (30.2)	9 (14.3)	8 (12.7)
2020	1,061	43 (4.1)	13 (1.2)	16.0 (11.0–24.0)	6 (14.0)	11 (25.6)	8 (18.6)	4 (9.3)
2021	1,141	50 (4.4)	16 (1.4)	24.0 (12.0–30.0)	9 (18.0)	19 (38.0)	9 (18.0)	7 (14.0)
*Z* value		2.639^c^	0.884^c^	2.528^d^	0.187^c^	−0.832^c^	−0.550^c^	−0.169^c^
*P* for trend		0.008	0.377	0.012	0.851	0.406	0.583	0.866

NEC, necrotizing enterocolitis; IQR, interquartile range.

a The denominator represents the number of NEC cases.

b NEC-related death was defined as death occurring within 7 days of NEC onset, in which NEC or its complications were considered very likely to have directly contributed to the death.

c Trend analysis was performed using the Cochran-Armitage test.

d Trend analysis was performed using the Jonckheere–Terpstra test.

Further stratified analysis showed that the incidence of NEC at gestational age < 28 weeks, 28–29 weeks, and 30–31 weeks was 7.7%, 5.8%, and 3.7%, respectively; and the incidence of NEC at birth weight < 1,000 g, 1,000–1,499 g, and ≥ 1,500 g was 8.3%, 5.3%, and 3.2%, respectively. With increasing gestational age and birth weight, the incidence of NEC, stage III NEC, and surgical treatment decreased (*P* for trend < 0.05), but the age at NEC onset occurred earlier (*P* for trend < 0.05). There was a temporal reduction in NEC incidence over the study period (*P* for trend=0.008).

### 3.3 Post-NEC clinical management

Among 156 NEC cases, 68.6% (107/156) infants received medical management while 31.4% (49/156) underwent surgical intervention (Table 3). In feeding management, the median duration of nil per os (NPO) and time to full enteral feeding were 8 (IQR: 5–11) and 25 (IQR: 14–38) days after NEC onset, respectively. Only 18.0% (28/156) of infants were initiated any human milk feeding after the NEC-related fasting period. For antibiotics management, the median duration of antibiotic therapy for NEC was 17 (IQR: 11–26) days, with carbapenems administered to 51.9% (81/156) of cases. Among the 24 infants with positive blood cultures within ±7 days of NEC onset, the isolated pathogens were as follows: Escherichia coli (9 cases, 37.5%), Klebsiella pneumoniae (8 cases, 33.3%), Staphylococcus capitis (4 cases, 16.7%), Staphylococcus epidermidis (2 cases, 8.3%), and Serratia marcescens (1 case, 4.2%). Regarding respiratory and circulatory management within the critical 3-day window post-NEC, blood transfusion, inotropic support, and invasive mechanical ventilation were required in 45.5% (71/156), 32.7% (51/156), and 44.2% (69/156) of cases, respectively, with significantly higher utilization in surgical versus medical management (all *P* < 0.001).

**Table 3:** Post-NEC clinical management.

Management	All NEC (*n* = 156)	Medical NEC(*n* = 107)	Surgical NEC (*n* = 49)	*P* value
Feeding management				
NPO duration post-NEC, days, median (IQR)^a^	8 (5–11)	8 (5–11)	9 (5–13)	0.660
Initiation of human milk feeding post-NEC, *n* (%)^b^	28 (18.0)	21 (19.6)	7 (14.3)	0.420
Days to full enteral feeding post-NEC, days, median (IQR)^c^	25 (14–38)	24 (14–35)	31 (17–40)	0.164
Antibiotic management				
Duration of antibiotic therapy for NEC, days, median (IQR)^d^	17 (11–26)	16 (10–25)	18 (11.5–36)	0.217
Antibiotic use at NEC onset				
Carbapenems, *n* (%)	81 (51.9)	48 (44.9)	33 (67.3)	0.009
Penicillins, *n* (%)	30 (19.2)	26 (24.3)	4 (8.2)	0.018
Cephalosporins, *n* (%)	24 (15.4)	22 (20.6)	2 (4.1)	0.008
Vancomycin, *n* (%)	21 (13.5)	11 (10.3)	10 (20.4)	0.085
Linezolid, *n* (%)	21 (13.5)	15 (14.0)	6 (12.2)	0.763
Aminoglycosides, *n* (%)	1 (0.6)	0 (0.0)	1 (2.0)	0.314
Circulatory management				
Red blood cell transfusion within 3 days of NEC onset, *n* (%)	71 (45.5)	38 (35.5)	33 (67.3)	<0.001
Inotropic drug requirement within 3 days of NEC onset, *n* (%)	51 (32.7)	22 (20.6)	29 (59.2)	<0.001
Respiratory management				
Invasive ventilation requirement within 3 days of NEC onset, *n* (%)	69 (44.2)	35 (32.7)	34 (69.4)	<0.001

NEC, necrotizing enterocolitis; NPO, nil per os; IQR, interquartile range.

a Time from NEC onset to successful enteral feeding resumption.

b Initiated any human milk feeding after the NEC-related fasting period.

c Among 130 NEC survivors, time from NEC onset to full enteral feeding establishment.

d Among 130 NEC survivors, time from NEC onset to antibiotic discontinuation (restarts ≤3 d included).

### 3.4 Clinical outcomes

Among 156 NEC cases, the overall in-hospital mortality was 16.7% (26/156), decreasing with advancing gestational age: 30% at < 28 weeks, 15.3% at 28–29 weeks, and 8.8% at 30–31 weeks. (Table 2). Compared with non-NEC controls, the NEC group had significantly higher mortality (16.7% vs. 8.2%), increased incidence of moderate-to-severe BPD (39.7% vs. 19.8%), and prolonged durations of both parenteral nutrition [36 [IQR:25–51] vs. 19 [IQR: 13–28] days] and hospitalization [54 [IQR: 39–70] vs. 42 [IQR: 31–55] days]. These associations remained significant after adjusting for key patient-level factors and accounting for center cluster effects using generalized estimating equations (all *P* < 0.05) (Table 4). Notably, clinical outcomes showed minimal differences between the medical and surgical treatment groups (Supplementary Table 1).

**Table 4:** NEC versus non-NEC on clinical outcomes in very preterm infants.

Outcomes	Non-NEC (*n* = 2,932), *n* (%)	NEC (*n* = 156), *n* (%)	Crude odds ratio/mean difference (95% CI)^a^	*P*	Adjusted odds ratio/mean difference (95% CI)^b^	*P*
Mortality	241 (8.2)	26 (16.7)	2.23 (1.44–3.47)	<0.001	1.61 (1.16–2.23)	0.004
BPD^c^	560/2,829 (19.8)	54/136 (39.7)	2.67 (1.86–3.81)	<0.001	2.03 (1.45–2.85)	<0.001
PVL^d^	85/2,756 (3.1)	6/148 (4.1)	1.33 (0.57–3.09)	0.510	0.80 (0.47–1.37)	0.413
ROP≥3^e^	231/2,268 (10.2)	18/116 (15.5)	1.62 (0.96–2.73)	0.070	0.80 (0.45–1.43)	0.453
EUGR^f^	996/2,691 (37.0)	69/130 (53.1)	1.93 (1.35–2.74)	<0.001	1.50 (0.69–3.29)	0.306
Duration of parenteral nutrition, days, median (IQR)^f^	19 (13–28)	36 (25–51)	16 (14–19)	<0.001	13 (11–15)	<0.001
Duration of hospitalization, days, median (IQR)^f^	42 (31–55)	54 (39–70)	11 (8–14)	<0.001	4 (2–6)	0.001

NEC, necrotizing enterocolitis; BPD, bronchopulmonary dysplasia; PVL, periventricular leukomalacia; ROP, retinopathy of prematurity; EUGR, extrauterine growth restriction; IQR, interquartile range.

a Univariate analysis using generalized estimating equations (GEE).

b Adjust for gestational age, male, multiple births, small for gestational age, inborn, 5-min Apgar ≤3, TRIPS score, antenatal steroids, rupture of membranes >24 h, and center cluster effect using generalized estimating equation (GEE).

c BPD was defined as ventilation or oxygen dependency at 36weeks’ postmenstrual age or at discharge, or death if before 36 weeks. Respiratory support data on 123 infants were not available.

d Among infants with neuroimaging results. neuroimaging results on 184 infants were not available.

e Among infants with ROP screening. ROP screening data on 704 infants were not available.

f Among surviving infants.

## 4 Discussion

This multicenter cohort study from China provided a comprehensive description of the epidemiology, clinical management, and outcomes of NEC in VPIs. We found that the overall NEC incidence (5.1%) and mortality (16.7%) were comparable to high-income country benchmarks; however, compared with these benchmarks, the 28–31 week subgroup showed higher incidence, whereas the <28 week subgroup showed higher mortality. Additionally, we observed several clinical findings that suggest potential targets for improvement, including perinatal care, human milk feeding, and antibiotic stewardship. These data provide regional evidence on the burden and management of NEC among VPIs in China and may help identify potential areas for quality improvement in prevention and care.

With an NEC incidence rate of 5.1%, our VPIs fall within the mid-range reported for high-income countries in recent years (2%–7%) (5, 6). However, this rate remains higher than those documented in Canada (4%) and Australia (4.6%) (6, 20). Compared to the Canadian Neonatal Network (CNN), the NEC incidence in the < 28 week subgroup was similar to that in Canada (7.7% vs. 7.9%) (20). Caution is warranted when interpreting this finding in the < 28-week subgroup. The survival rate of infants < 28 weeks in China remains significantly lower than in developed countries (21). Some infants died before reaching the high-risk period for NEC, potentially leading to an underestimation of the incidence in this subgroup, which may partially explain the comparable rates between our cohort and Canada.

In contrast, the NEC incidence in 28–31 week subgroup was significantly higher than in Canada (4.5% vs. 2%) (20). The elevated incidence may be related to multiple potentially modifiable factors. First, our human milk feeding rate was substantially lower than that in Canada (52.1% in Table 1 vs. 78.6%) (22, 23). This disparity is clinically significant given the robust evidence that human milk feeding reduces NEC risk —with mother's own milk offering the greatest protection (24–27). These findings suggest that strengthening regional systems to support mother's own milk feeding may be an important area for future quality-improvement efforts in VPIs in China. In addition, this study revealed lower rates of both antenatal steroids administration (78.1% in Table 1 vs. 89.8%) and delayed cord clamping (17.7% in Table 1 vs. 61.4%) compared to the CNN data (20). Both interventions are established protective factors against NEC in VPIs (28, 29). Thus, we need to implement multidisciplinary quality improvement programs involving obstetrics and neonatology to enhance standardized perinatal management. At the same time, further studies incorporating temporally defined pre-NEC clinical characteristics are needed to better understand the relatively higher NEC incidence among infants born at 28–31 weeks.

This study identified a median NEC onset at 18.0 (IQR: 10.0–28.5) days postnatal age. Notably, we observed that gestational age was inversely correlated with the timing of onset, which aligns with previous studies (30–32). These results delineate the time window for NEC onset across different gestational age subgroups. Interestingly, although the age at onset varied by gestational age, the corrected gestational age at NEC onset generally clustered in the critical window of 31–32 weeks. This finding suggests that corrected gestational age, rather than postnatal age, may be a more accurate predictor of NEC onset, providing a crucial temporal reference for clinical monitoring.

Additionally, the median duration of post-NEC antibiotic therapy in our cohort was 17 (IQR: 11–26) days, and carbapenems were administered to 51.9% of cases. However, detailed treatment indications, antimicrobial susceptibility data, and center-specific antibiotic protocols were unavailable, limiting our ability to assess the appropriateness of antibiotic selection and duration. These findings suggest that antibiotic stewardship may represent a potential area for future quality-improvement efforts.

The overall NEC mortality rate of 16.7% in our VPIs is at the lower range of global reports (15%–30%) (31, 32). Compared to the CNN data, our cohort showed comparable mortality rates in the 28–31 week subgroup (12.1% vs. 15.2%), but significantly higher mortality in the < 28 weeks group (30% vs. 20.8%) (20). These findings underscore the critical need for improved management strategies, particularly for infants born at < 28 weeks’ gestation. In addition, this study revealed significantly higher mortality and complication rates in NEC infants compared to non-NEC infants. While further stratified or causal analyses were not performed due to the descriptive and observational nature of our study, the results provide a descriptive assessment of mortality differences after adjusting for key patient-level factors. Therefore, proactive NEC prevention is essential to reduce severe complications and improve survival and quality of life. Notably, we observed no significant differences in mortality or the majority of complications between the medically and surgically managed NEC cases. Although we adjusted for key patient-level factors, Bell stage was not directly included in the analysis, potentially affecting the precision of severity adjustment between groups. In addition, the relatively small sample size of the surgical NEC group may have limited statistical power, and selection bias cannot be fully excluded. Therefore, these findings should be interpreted with caution. Although no major differences in outcomes were observed between the medical and surgical groups, the limited sample size and potential residual confounding preclude definitive conclusions regarding the comparative impact of surgical intervention on outcomes.

To our knowledge, few multicenter regional studies from China have described NEC epidemiology together with post-NEC management patterns and short-term outcomes among VPIs. By providing data on incidence, gestational-age-specific patterns, clinical management, and outcomes, this study helps fill an important regional knowledge gap and may help identify potential areas for quality improvement in NEC prevention and care. However, the study has several limitations. First, the relatively small number of NEC cases across centers limited our ability to assess inter-center variability. Second, because of the observational design, our management-related findings should be interpreted as descriptive. In particular, some in-hospital course measures, including time to full enteral feeding, invasive ventilation, red blood cell transfusion, and inotropic support, may have occurred after NEC onset and therefore should not be interpreted as antecedent risk factors for NEC. Although the multivariable GEE models adjusted for several important patient-level factors and center clustering, residual or unmeasured confounding cannot be excluded. Third, some outcome assessments were incomplete, particularly ROP screening and neuroimaging. Analyses of these outcomes were based on available cases, and missing data may have introduced selection bias and reduced the precision of the estimates. Fourth, the NICU-based rather than population-based nature of our cohort means that our findings may not fully represent the population-level epidemiology of NEC in China.

## 5 Conclusion

In this regional multicenter cohort from Jiangsu, China, the overall incidence and mortality rates of NEC among VPIs were comparable to high-income country benchmarks. However, gestational-age-stratified comparisons revealed higher incidence in the 28–31 week subgroup and higher mortality in the <28 week subgroup compared with these benchmarks. These findings highlight the importance of gestational-age-stratified interpretation and suggest that future quality-improvement efforts in similar NICU settings should address the excess NEC incidence among infants born at 28–31 weeks and the excess mortality among those born at <28 weeks. Potential areas for such quality-improvement efforts include perinatal care, human milk feeding, and antibiotic stewardship. These quality-improvement priorities may also be relevant to other developing countries.

## Data Availability

The raw data supporting the conclusions of this article will be made available by the authors, without undue reservation.
