# Supplementary material for: Necrotizing enterocolitis in very preterm infants: an epidemiological study in Jiangsu, China
Source: Front Pediatr. 2026 Sep 23;14:1948550. doi: 10.3389/fped.2026.1948550 (PMC13642806; doi:10.3389/fped.2026.1948550)
Supplement: Supplementary file 1 [file Supplementaryfile1.docx]

**Supplementary Material File**

Contents

[Suppl Figure 1: Study population.	2](#_Toc19554)

[Suppl Table 1 Medical NEC versus surgical NEC on clinical outcomes.	3](#_Toc6526)

**Suppl Figure 1: Study population.**

Abbreviations: NICUs, neonatal intensive care units; NEC, Necrotizing enterocolitis.

**Suppl Table 1** Medical NEC versus surgical NEC on clinical outcomes.

| **Outcomes** | **Medical NEC (n=107), n (%)** | **Surgical NEC (n=49), n (%)** | **Crude Odds Ratio/Mean Difference (95% CI)**^a^ | ***P*** | **Adjusted Odds Ratio/Mean Difference (95% CI)**^b^ | ***P*** |
| --- | --- | --- | --- | --- | --- | --- |
| Mortality | 15 (14.0) | 11 (22.4) | 1.78 (0.75-4.22) | 0.193 | 1.51 (0.41-5.57) | 0.533 |
| BPD^c^ | 34/98 (34.7) | 20/38 (52.6) | 2.09 (0.98-4.47) | 0.057 | 2.16 (1.16-4.01) | 0.015 |
| PVL^d^ | 5/101 (5.0) | 1/47 (2.1) | 0.42 (0.05-3.68) | 0.432 | 0.43 (0.11-1.65) | 0.220 |
| ROP≥3^e^ | 13/83 (15.7) | 5/33 (15.2) | 0.96 (0.31-2.95) | 0.945 | 0.87 (0.36-2.10) | 0.754 |
| EUGR^f^ | 44/92 (47.8) | 25/38 (65.8) | 2.10 (0.96-4.60) | 0.064 | 1.66 (0.62-4.42) | 0.309 |
| Duration of parenteral nutrition, days, median (IQR)^f^ | 34.5 (25-47) | 39 (27-55) | 6 (-1-13) | 0.079 | 2 (-10-15) | 0.688 |
| Duration of hospitalization, days, median (IQR)^f^ | 54 (42.5-67) | 54.5 (31-82) | 2 (-7-11) | 0.685 | -3 (-16-10) | 0.657 |

Abbreviations: NEC, necrotizing enterocolitis; BPD, Bronchopulmonary dysplasia; PVL, Periventricular leukomalacia; ROP, Retinopathy of Prematurity; EUGR, Extrauterine growth restriction.

^a^Univariate analysis using generalized estimating equations (GEE).

^b^Adjust for gestational age, male, multiple births, small for gestational age, inborn, 5-min Apgar ≤3, TRIPS score, antenatal steroids, rupture of membranes >24 h, and center cluster effect using generalized estimating equation (GEE).

^c^BPD was defined as ventilation or oxygen dependency at 36weeks’ postmenstrual age or at discharge, or death if before 36 weeks. Respiratory support data on 20 infants were not available.

^d^Among infants with neuroimaging results. neuroimaging results on 8 infants were not available.

^e^Among infants with ROP screening. ROP screening data on 40 infants were not available.

^f^Among surviving infants.
